# Tribological Behaviors of Graphene and Graphene Oxide as Water-Based Lubricant Additives for Magnesium Alloy/Steel Contacts

**DOI:** 10.3390/ma11020206

**Published:** 2018-01-29

**Authors:** Hongmei Xie, Bin Jiang, Jiahong Dai, Cheng Peng, Chunxia Li, Quan Li, Fusheng Pan

**Affiliations:** 1 College of Mechanical and Electrical Engineering, Yangtze Normal University, Chongqing 408100, China; xiehongmei@yznu.cn (H.X.); 20091008@yznu.cn (J.D.); 2 College of Materials Science and Engineering, National Engineering Research Center for Magnesium Alloys, Chongqing University, Chongqing 400044, China; fsPan@cqu.edu.cn; 3 Chongqing Academy of Science and Technology, Chongqing 401123, China; liquan@cqu.edu.cn

**Keywords:** graphene, graphene oxide, water-based, lubricant additives, magnesium alloy

## Abstract

The tribological behaviors of graphene and graphene oxide (GO) as water-based lubricant additives were evaluated by use of a reciprocating ball-on-plate tribometer for magnesium alloy-steel contacts. Three sets of test conditions were examined to investigate the effect of concentration, the capacity of carrying load and the endurance of the lubrication film, respectively. The results showed that the tribological behaviors of water can be improved by adding the appropriate graphene or GO. Compared with pure deionized water, 0.5 wt.% graphene nanofluids can offer reduction of friction coefficient by 21.9% and reduction of wear rate by 13.5%. Meanwhile, 0.5 wt.% GO nanofluids were found to reduce the friction coefficient and wear rate up to 77.5% and 90%, respectively. Besides this, the positive effect of the GO nanofluids was also more pronounced in terms of the load-carrying capacity and the lubrication film endurance. The wear mechanisms have been tentatively proposed according to the observation of the worn surfaces by field emission scanning electron microscope-energy dispersive spectrometer (FESEM-EDS) and Raman spectrum as well as the wettability of the nanofluids on the magnesium alloy surface by goniometer.

## 1. Introduction

Magnesium and its alloys are attractive materials for a wide range of applications in such demanding fields as transportation, electronics or aerospace [1,2]. This is due to superior attributes such as low density, high thermal conductivity, and ease of manufacturing by conventional processes. Currently, most of the magnesium alloy usage in industry is limited to die-cast components [3]. In the future, the increasing demand for large-scale applications of wrought Mg alloy products, such as extruded profiles, rolled sheets and forgings, will determine the development of Mg alloys. The wrought Mg alloys offer better mechanical properties in comparison to cast Mg alloys because of the pronounced grain refinement without pores and uniform composition distribution after the deformation process [4]. However, the high friction between the workpiece and tool steel during the forming process cannot be avoided, thus resulting in relatively short tool life, high energy consumption and poor-quality products [5,6].

To address these issues, lubricants can be employed. So far, there are no suitable forming fluids for the forming process of Mg alloy; even in some conditions, the forming fluid used for Al alloy is casually used and the result is not satisfied. The commonly used Al alloy forming lubricants relies heavily on sulfur-, chlorine- and phosphorous-containing organic compounds as additives. The discharge of these lubricants has caused a series of issues such as high cost, environmental pollution and others [7]. Furthermore, the effects of these additives on tribological properties can be complicated by the fact that the fast chemical degradation during friction process is often inevitably present in them and degrades their desired tribological behaviors [8]. Therefore, a new type of environmentally sustainable lubricant additives that do not compromise lubricant performances needs to be required to substitute the conventional additives. To ameliorate the tribological performances of magnesium alloys, several categories of advanced additives have been explored, such as N-containing compounds [9], borates [10] and ionic liquids [11]. Overall, these lubricant additives have exhibited excellent tribological properties during testing, mainly because of the formation of different types of tribofilm between the interacting surfaces. Although many nitrogen heterocyclics showed favorable wear resistance and corrosion inhibition, the friction reduction performance was limited [12]. The borate without active element, such as nitrogen, sulfur and chlorine, is ineffective at improving the tribological properties of magnesium alloy [13]. The ionic liquids are synthesized from expensive starting material, which results in the high cost to use these ionic liquids as lubricant additives [14]. Therefore, the search continues for novel materials that can potentially be used as lubricant additives for magnesium alloys.

Lately, a wide variety of nanomaterials as lubricant additives is a rapidly progressing field of research owing to their unique chemical and structural attributes [15,16,17]. Compared with conventional organic lubricant additives, the nanoparticles as lubricant additive possess several advantages; for example the small size allows the nanoparticles to readily enter the tribological interfaces [18]. More importantly, the excellent chemical stability of the nanoparticles contributes to less harmful emissions and dramatically lower toxicity than conventional organic additives, making them attractive with respect to the sustainable development of environmental. Among a variety of nanoparticles, the two-dimensional graphene and GO possess the desirable properties from a lubrication point of view, such as extreme strength, easy shear capability, excellent Young’s modulus, high thermal stability and good conductivity, and have been extensively explored for their tribological behaviors as self-lubricating solids [19,20], as composites [21,22,23] and as lubricant additives. For example, Harshal P. Mungse et al. [24] evaluated the tribological properties of graphene oxide sheets as additives in conventional 10W-40 lube oil for steel/steel pairs. It was reported that GO nanosheets, used as a lubricant oil additive, played a positive role in remarkably lowering the friction and improving the anti-wear properties. The friction coefficient and wear volume were reduced by about 36.4% and 37.5% respectively. Yinglei Wu et al. [25] used a ball-on-ring tribometer to investigate the tribological behavior of GO sheets, which were dispersed in O/W base emulsion. Their results revealed 27.9% and 21.8% reductions in friction coefficient and wear volume, respectively, attributed to the formation of a GO-related lubricating tribofilm on the worn surface. Xiaoqiang Fan et al. [26] reported the use of graphene as a bentone-lubricating grease additive for the steel/steel pairs. The experimental measurements showed that friction-reducing and anti-wear properties of bentone-lubricating grease were both improved by the formation of a graphene-related lubricating boundary layer. Nevertheless, no improvement of the lubricating behaviors was observed for the copper alloy/steel contacts when graphene was dispersed into the hydraulic oil [27]. Previous studies demonstrate that the lubrication effectiveness of GO and graphene as additives are very sensitive to the tribological system properties such as based media and contact pairs. Clearly, water-based lubrication processes the advantage of cooling capabilities, reduced cleaning costs, lower toxicity and fire resistance. However, the low viscosity, corrosive properties and poor boundary lubrication properties limit the application of water lubrication in the process of metal forming, and extra additives are necessary for enhancing its lubricating properties. To date, the combination of graphene or GO water-based fluids and magnesium alloy are still waiting to be explored.

The aim of this study was to evaluate the tribological performances of graphene and GO as water-based lubricant additives for the magnesium alloy/steel contacts using a ball-on-plate tribotester. The lubrication mechanism was investigated by analyzing the worn surface using FESEM-EDS and Raman spectroscopy. It is anticipated that graphene or GO water-based nanofluids may find broad potential applications in the forming process of magnesium alloy.

## 2. Experimental

### 2.1. Materials

**Figure 1:**
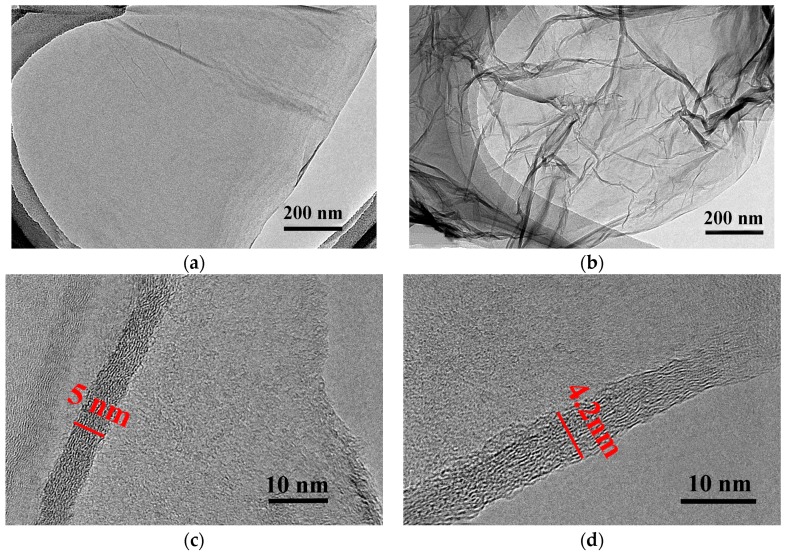

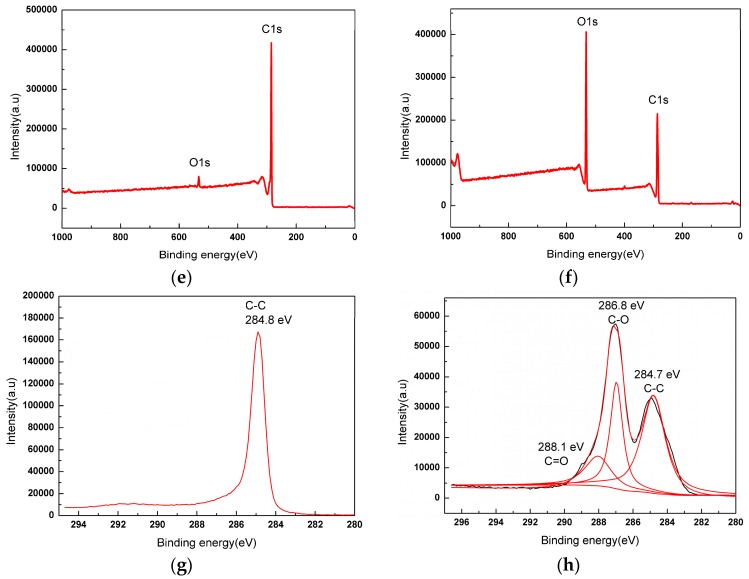
TEM images and XPS spectrum of graphene (**a**,**c**,**e**,**g**) and GO (**b**,**d**,**f**,**h**).

The graphene and GO used in this work were commercially obtained from Hengqiu Graphene technology Co., Ltd., Suzhou, China. The morphology and structure of the samples were observed with JEM 1200EX transmission electron microscope (TEM; JEOL Ltd., Tokyo, Japan) and VG model Escalab 250 X-ray photoelectron spectroscopy (XPS, VG Scientific Ltd., East Grinstead, UK). As shown in TEM images (Figure 1a,b), graphene is apparently transparent with the size of several micrometers, while the GO nanosheets with many folds are observed. These rippled morphologies can be attributed to the oxygen-containing groups on the GO nanosheets. These oxygen containing groups attracted to each other through hydrogen bond, resulting in folds in GO sheets. The high-resolution transmission electron microscope (HRTEM, JEM 1200EX, JEOL Ltd., Tokyo, Japan) lattice image as shown in Figure 1c,d clearly demonstrated that the thickness of graphene sheets and GO was 5 nm and 4.2 nm, respectively. Based on the reported interlayer distances for graphene and GO layers (0.34 nm and 0.7 nm), the graphene and GO were identified to be multi-layer [28]. XPS spectrum (Figure 1e,f) is demonstrated the difference in the carbon composition for graphene and GO, as well as to emphasize the oxidation level of GO. The C1s spectrum of GO as shown in Figure 1 consists of three different peaks: C–C in aromatic rings (284.7 eV), C–O (286.8 eV) and C=O (288.1 eV), indicating the existence of these oxygen-containing functional groups. The nanofluids were prepared by mixing the given additives (graphene and GO) into the water at different concentration i.e., 0.2, 0.5, 0.7 and 1.0 wt.%. In order to obtain a uniform mixture, the suspensions were stirred for 1 h, followed by ultrasonic bathing for 2 h. In this study, any extra dispersion or surfactant agents were not used so as to isolate the effects of the nanoparticles additives.

The commercial AZ31 (Mg–3.07Al–0.78Zn–0.38Mn in wt.%) magnesium alloy ingots with 82 mm in diameter were homogenized at 380 °C for 2 h, and then were extruded to plates with dimensions of 56 mm in width and 3 mm in thickness at 380 °C. Several tensile tests were performed by a CMT6305-300 KN universal testing machine (Skyan power equipment Ltd., Shenzhen, China) with an initial speed of 1.5 mm·min^−1^ at room temperature. In tensile test, at least three samples were tested for each condition and the average value was then obtained. The mechanical behaviors of the extruded AZ31 magnesium alloy are presented in Table 1. The as-extruded plates were then divided into samples with dimensions of 20 mm (Extrusion direction) × 10 mm (Transverse direction) × 3 mm (Normal direction). Prior to friction test, the samples were successively polished with 600 and 1000 grit silicon carbide paper, and then ultrasonic degreased in alcohol. The arithmetic average surface roughness (Ra) of the specimens tested was measured as 0.08 μm using the Olympus OLS4000 3D surface mapping profilometer (Olympus Ltd., Tokyo, Japan).

### 2.2. Tribological Tests

**Figure 2:**
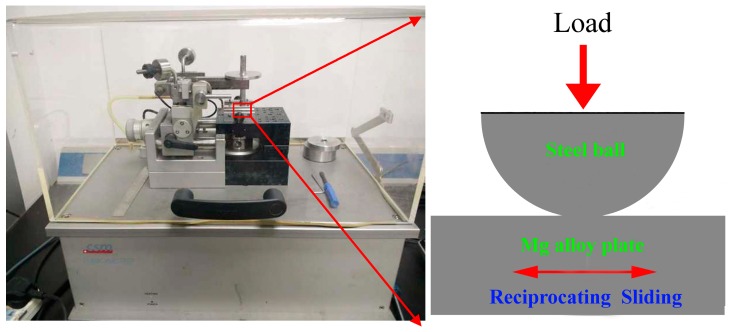
The ball-on-flat reciprocating sliding tester and schematic of ball-pot assembly in ball-on-flat tribotester.

The tribological properties of graphene and GO as water-based additives for AZ31 magnesium alloy/AISI 52100 steel contacts were investigated on a reciprocating tribometer with a ball-on-plate configuration (CSM Instruments, Peseux, Switzerland) in the ambient environment. Experimental set up of the tribotester is presented in Figure 2 along with schematic view of ball-on-plate assembly. The reference gives a detailed introduction to the principle of the tribometer [29]. The prepared AZ31 magnesium alloy was used as the stationary plate specimen while 6 mm diameter steel ball (hardness 690 HV_0.01_, surface roughness Ra 0.05 μm) was sliding with a speed controlled by a DC servomotor. Three sets of test conditions (shown in Table 2) were examined to evaluate the effect of concentration, the capacity of carrying load and the endurance of the lubrication film, respectively. For the influence of nanoparticle concentration on lubrication performance, graphene and GO as additives dispersed into water with different mass concentration of 0, 0.2, 0.5, 0.7 and 1.0 wt.%, respectively, were investigated. These concentration values were established from the results of bibliographic research, where concentration of graphene or GO was in the range of 0.1–1 wt.% [30,31]. Tests were conducted by applying a normal load of 3 Nat speed of 0.08 m/s with a 6 mm stroke for a rubbing time of 30 min. The Hertzian contact stress at the beginning of the test is calculated to be 312 MPa, which is at least 30% higher than the yield strength of magnesium alloy sheets. The optimal concentration of graphene or GO dispersed in the water was obtained from the above-mentioned tests. For the load-carrying capacity test, four different normal loads (1, 3, 5, 8 N) were applied to the upper stationary steel ball which slid at 0.08 m/s against the lower magnesium alloy plate for 30 min. Finally, some long lasting wear tests were carried out by applying 8 N of normal load at 0.03 m/s sliding speed. This set of testing conditions was chosen to evaluate the endurance of the lubrication film. During the sliding process, the friction coefficient was recorded in situ and a sudden increase in the friction coefficient was regarded as lubrication-film breakdown. The lubricant was applied onto the plates as droplets with a pipette before operation. The droplets formed a continuous lubricating layer covering the entire wear track area. At least three replicates were run for each lubricant and the final results of the friction coefficient and wear rate shown in this study were the average value.

### 2.3. Surface Characterization

The plate samples were cleaned in an alcohol ultrasonic bath for 5 min to eliminate the contaminants prior to the surface analysis. The rubbing surfaces on the magnesium alloys were analyzed by Zeiss AURIGA field emission scanning electron microscope (SU8010, Hitachi Ltd., Tokyo, Japan) integrated with energy dispersive spectrometer (FESEM-EDS; ESCA+, OXFORD instrument Ltd., London, UK). The Raman spectroscopy (Lab RAM HR800, with 532 nm laser excitation; Horiba Jobin Yvon S.A.S Ltd., Pairs, France) of the wear tracks on the plates was used to analyze the characteristics of the graphene or GO residue. The wettability of the sliding counterparts was evaluated by determining the static contact angle of pure water and nanofluids droplets on the magnesium alloy surface (Ra ~ 0.08 μm) using JC-2000C1 goniometer (Zhongchen Digital Technic Apparatus Ltd., Shanghai, China). A 5 μL volume droplet was placed on the magnesium alloy surface using a syringe, and the reported values in this study were reproducible for five identical samples. The wear volume of the lower magnesium alloy plate was measured by Olympus OLS4000 laser scanning confocal microscope (Olympus Ltd., Tokyo, Japan). Three replicates of friction and wear measurements were performed to minimize data scattering. In addition, then, the wear rate *w* was estimated by Equation (1):

(1) $$w=\frac{V}{F_{N}\times S}$$

where *V* = wear volume (mm^3^), *F_N_* = applied normal load (N), *S* = slidingdistance (m).

## 3. Results and Discussion

### 3.1. Influence of Nanoparticle Concentration on Lubrication Performance

**Figure 3:**
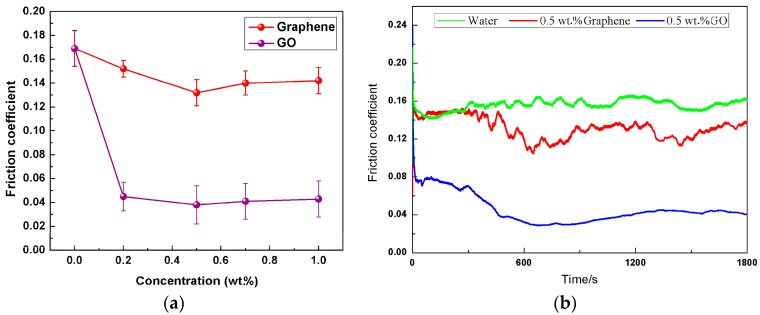
(**a**) The average friction coefficientas a function of nanoparticles concentration in water and (**b**) the friction coefficient relative to sliding time (3 N, 0.08 m/s, 30 min).

It is well known that the concentration of nanoparticles in the base lube plays a predominant role to control the friction coefficient and wear rate [32]. In this study, the effect of graphene and GO concentrations in the water ranging from 0.2 to 1.0 wt.% on the tribological properties was performed for magnesium alloy/steel contacts under 0.08 m/s at the load of 3 N for 30 min test duration. The pure water lubrication was chosen as a benchmark compared with the lubrication effects of nanofluids. The average friction coefficient as a function of nanoparticles concentration in water is displayed in Figure 3a. The correlation between friction coefficient and sliding time is given in Figure 3b. Accordingly, testing the pure water produced a high maximum friction coefficient and large fluctuations about the friction coefficient curve persisted. The main reason for this is that the low viscosity and pressure viscosity index of water was hard to form a sufficiently thick lubrication film between the contact interfaces. Both graphene and GO dispersed in water at the concentration of 0.5 wt.% showed best friction reducing in contrast with those at lower and higher concentrations due to negative lubrication effects caused by insufficient or excess amounts of graphene or GO in the contact area. Meanwhile, the effect of GO as lubricant additive on friction reducing was particularly noticeable. The 0.5 wt.% GO addition into the water reduced the friction coefficient by 77.5% (from 0.169 to 0.038 in average value) when compared with the pure water. Instead, there was slight improvement in the friction-reducing ability of the water in the presence of graphene, which reduced the value of friction coefficient by only 21.9% (from 0.169 to 0.132 in average value). In addition, it needs to be emphasized that the friction coefficient of the 0.5 wt.% graphene nanofluids during the initial few minutes of the sliding time has little difference from that of the pure water. Upon further increasing sliding time, the friction coefficient gradually drops. It can be inferred that the graphene additive in the water have not taken any effect on the friction coefficient at the early stage of the test, the water plays the major role between the rubbing pairs. Compared with 0.5 wt.% grapheme nanofluids, the high initial friction coefficient value was eliminated when 0.5 wt.% GO nanofluids were used. Moreover, the 0.5 wt.% graphene oxide nanofluids exhibited stability and consistent low friction coefficient within overall sliding time, which indicated a more positive effect from the friction reducing throughout the testing process. 

**Figure 4:**
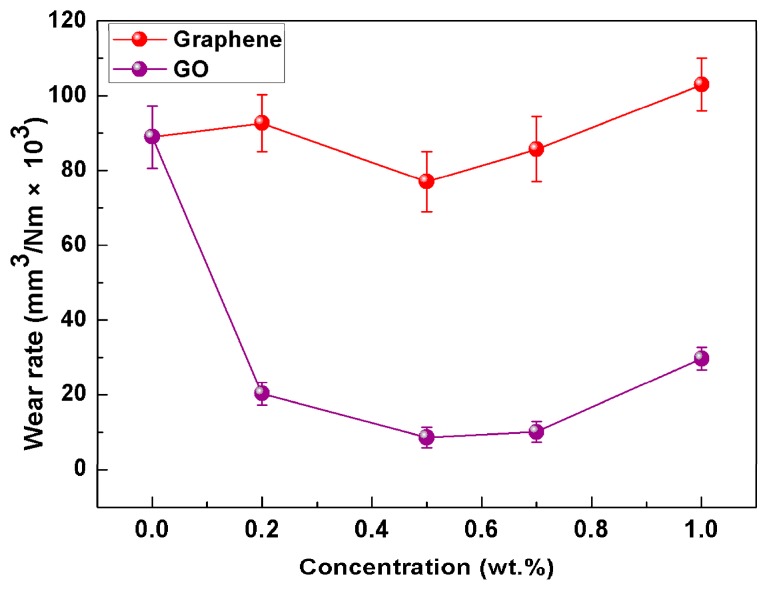
The average wear rate as a function of nanoparticles concentration in water (3 N, 0.08 m/s, 30 min).

**Figure 5:**
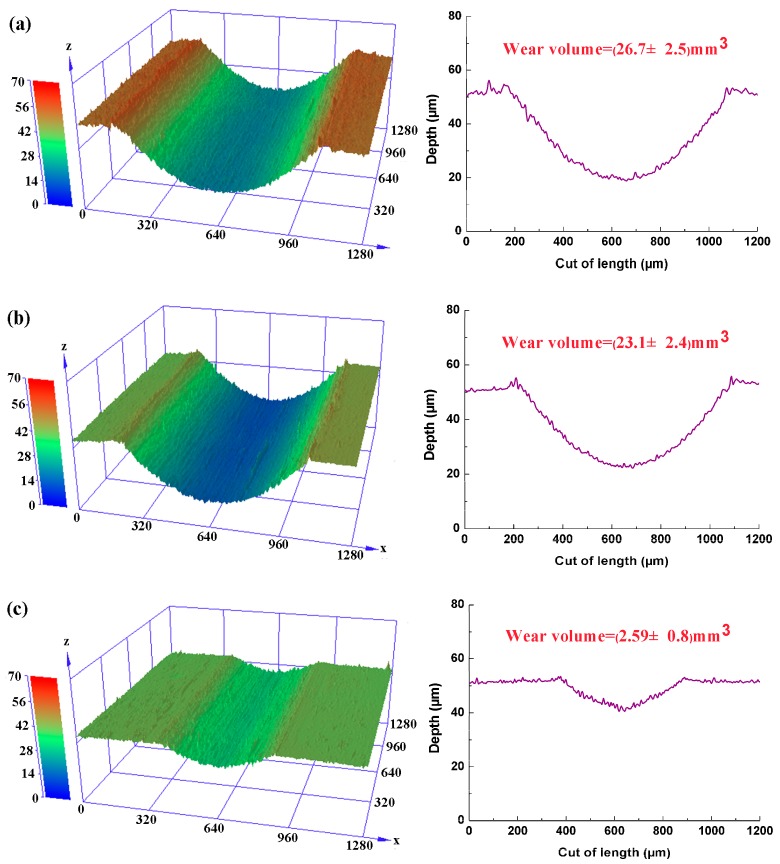
3D topography images and 2D profiles across the wear tracks for flat specimens after wear tests with (**a**) pure water; (**b**) 0.5 wt.% graphene nanofluids; (**c**) 0.5 wt.% GO nanofluids (3 N, 0.08 m/s, 30 min).

For a prospective lubricant to be considered effective, it is also necessary to safeguard the underlying surface from wear and damage. The steel ball experienced little wear because its hardness is much greater than that of the magnesium alloy plates. Therefore, the worn scars on the AZ31 magnesium alloy plate were mainly analyzed in the present study. The variation of wear rate in Figure 4 reveals similar change in the friction coefficient. The best wear resistance results found at 0.5 wt.%, and smaller improvements at higher concentration (e.g., 0.7 wt.%, 1.0 wt.%). When the concentration of nanoparticles is too high, the redundant nanoparticles will aggregate, which limits the amount of nanoparticles from supply zone to transition zone and further hinders the formation of tribofilm on the worn surfaces [33]. The addition of 0.5 wt.% graphene into the water reduced the wear rate by 13.5% compared with the pure water. In marked contrast, the wear rate lubricated with 0.5 wt.% GO nanofluids was nearly an order of magnitude smaller than that of pure water. These results demonstrated the beneficial contribution of graphene and GO in promoting anti-wear property of pure water, and the advantage of GO over graphene can be clearly seen. Figure 5 presents the 3D topography images and 2D profiles of the wear tracks on the plate. The wear track lubricated with pure water was obviously deeper than the tracks lubricated with the nanofluids. The optimal lubrication was 0.5 wt.% GO nanofluids, which was confirmed by the shallower wear tracks. According to demonstration research above, the optimized nanoparticles concentration in the water has been found to be 0.5 wt.%.

### 3.2. Influence of Normal Load on Lubrication Performance 

**Figure 6:**
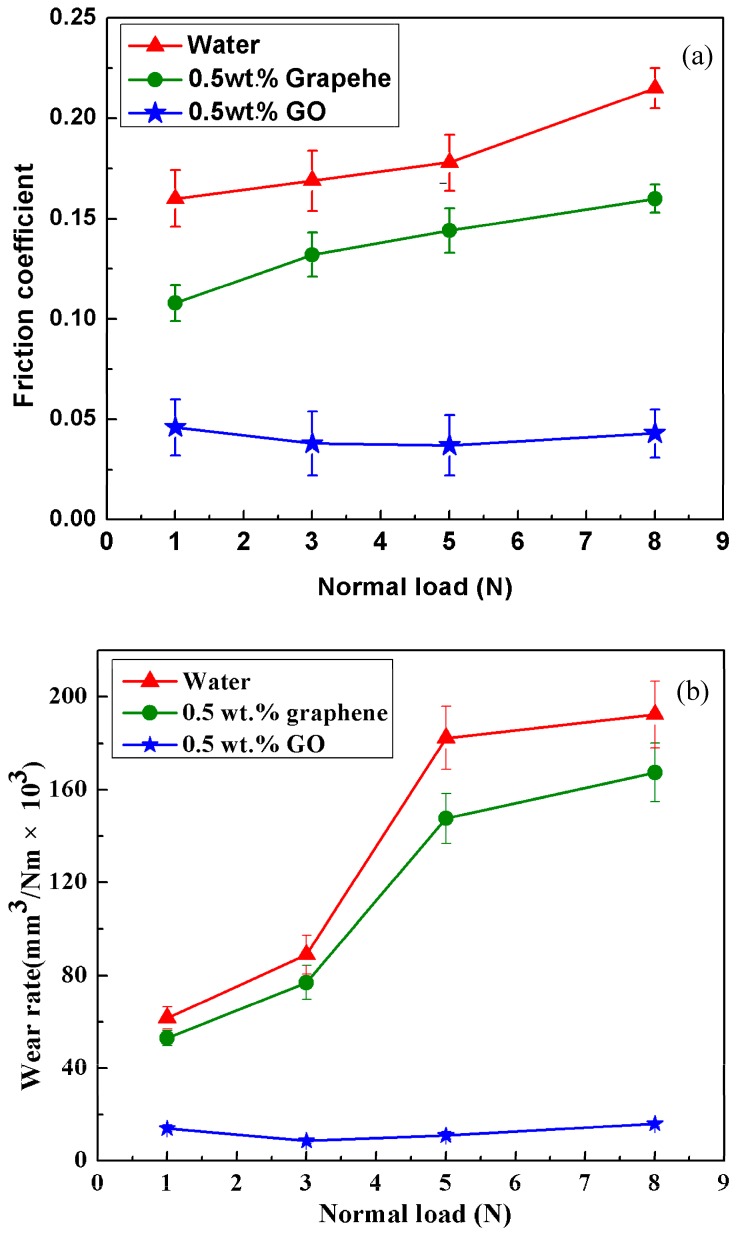
Effects of loads on average friction coefficient (**a**) and wear rate (**b**) of magnesium alloy specimens lubricated by the water with and without nanoparticles (0.08 m/s, 30 min).

The friction-reducing and anti-wear ability of 0.5 wt.% graphenenanofluids and 0.5 wt.% GO nanofluids for magnesium alloy/steel contacts were further investigatedin detail. Except for nanofluids, the pure water was chosen for comparison. The effect of load on the tribological properties of pure water and nanofluids are discussed and the results are shown in Figure 6. It can be seen that both the friction coefficient and wear rate for pure water and graphene nanofluids remarkably increased with increasing the normal load. Even so, the growth rate was quite different. The friction coefficient and wear rate for the 0.5 wt.% graphene nanofluids were smaller than that of pure water under each load. This may be ascribed to the fact that with an increase of the normal load, the micro-intervals between two rubbing pairs further decreased. In this case, there was less water at a molecular level that could be drawn into the sliding contact interface, resulting in significantly reduced lubrication property. As for the test of the graphene nanofluids, the graphene-based tribofilm forms on the rubbing surfaces can avoid the metal-metal contact interface [34]. Therefore, there is slight enhancement in the friction-reducing and anti-wear performances of the pure water in the presence of graphene. Even so, observation of the increase in friction coefficient and wear rate with load may be attributed to relatively thinner tribofilm formation and its removal under high load. Instead, the values of friction coefficient and wear rate are found to be much lower in the case of surface lubricated with GO nanofluids at the same operating load than the surface lubricated with graphene nanofluids. Moreover, both of the friction coefficient and wear rate for the GO nanofluids were constant for all the tested normal loads. This was ascribed to the stable existence and continuous protection of tribo-layer on the contacted surfaces. The possible tribological enhancing mechanisms for the GO nanofluids are further analyzed in the “Related tribological mechanism of nanofluids” section.

### 3.3. The Endurance of Lubrication Film

**Figure 7:**
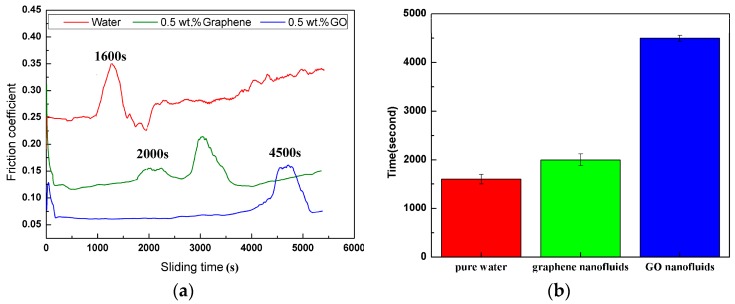
Sliding time to lubrication-film breakdown (**a**) and the average time of the lubrication films fails; (**b**) (8 N, 0.03 m/s, 90 min).

The stability of the lubrication film with time is a very important property in mechanical systems needed for their functionality during their service life. In order to evaluate the lubrication film endurance during sliding, the severity of the contact conditions was increased by increasing the applied normal load and sliding time and reducing the sliding velocities. Figure 7 summarizes the tribological results conducted under a normal load of 8 N and sliding speed of 0.03 m/s for 90 min test duration. As can be observed in Figure 7, the lubrication film could not survive for a long period of time in the case of pure water lubrication, and its friction coefficient abruptly increased to 0.35 after about 1600 s. It indicated the rupture of the lubrication film formed by pure water on the wear track, resulting in a break in the steady state. Adding 0.5 wt.% graphene into the water decreased the friction coefficient and prolonged the lubrication film’s stability to about 2000 s of sliding, which lasted approximately 1.25 times longer than that of the pure water. Though the graphene nanofluids can decreased the friction coefficient to some degree and protected the AZ31 Mg alloy temporarily, the durability and stability were not good enough. On the contrary, the GO nanofluids cannot only significantly decrease the friction coefficient, but also possess excellent lubrication film endurance. The graph shows that the durability of the GO nanofluids lasted 4500 s, which was approximately 2.8 times longer than that of the pure water. These results demonstrated the superior friction-reducing performance and lubrication endurable of the GO as water-based additive for magnesium alloy/steel pairs even under severe operating conditions.

### 3.4. Surface Wettability Test

**Figure 8:**
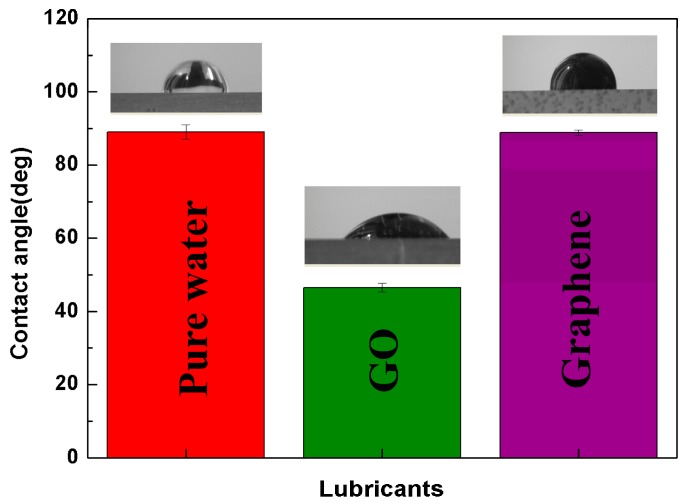
The contact angle of AZ31 magnesium alloy surface for pure water, 0.5 wt.% graphene nanofluids and 0.5 wt.% GO nanofluids.

The wetting of the water-based nanofluids on the metal surface is very critical to lubricant performances. Generally, the excellent wettability intends to promote the formation of a tribofilm, which can separate the asperity contact [28]. Figure 8 illustrates the contact angle data of pure water, 0.5 wt.% graphene nanofluids and 0.5 wt.% GO nanofluids on the polished magnesium alloy surface. It was observed that the contact angle for pure water was found to be 89°. The addition of GO in water has markedly reduced the contact angle from 89° to 46.5°. In contrast, the graphene nanofluids did not show obvious change in contact angle in contrast with pure water (89° versus 88°). Contact angle values provide information about the ability of lubricants to wet the metal surfaces and to interact with them to form surface films. In this study, the magnesium alloy surface is more wettable for the GO nanofluids in comparison with pure water and graphene nanofluids. Therefore, we can deduce that it is more facile for the GO nanofluids than the graphene nanofluids to form the protective layer due to their different wettability of the contact sliding interfaces. The obtained results are showing good agreement with the tribological performances.

### 3.5. The Worn Surface Analysis

**Figure 9:**
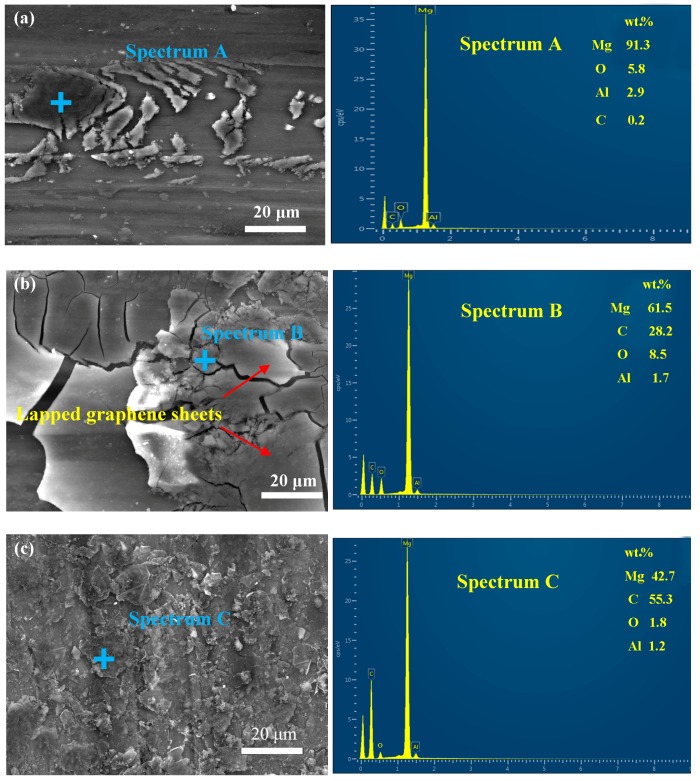
FESEM-EDS results for the wear surface lubricated with (**a**) pure water; (**b**) 0.5 wt.% graphene nanofluids; (**c**) 0.5 wt.% GO nanofluids (3 N, 0.08 m/s, 30 min).

Figure 9 exhibits the FESEM micro-morphologies and the corresponding EDS analysis of the worn surface lubricated by pure water, 0.5 wt.% graphene nanofluids and 0.5 wt.% GO nanofluids under 0.08 m/s at the load of 3 N for 30 min. As shown in Figure 9a, the worn surface lubricated with pure water showed signs of deep grooves and significantly desquamation from body surface, possibly because the water caused tribo-corrosion during the sliding. It was therefore reasonable to infer that this condition was dominated by abrasive wear and corrosion wear. Therefore, the pure water failed to act effectively under constant loading cycle. The corresponding EDS analysis in the spectrum A displayed that the content of C element on the worn surface was only 0.2 wt.%, which may be ascribed to the pollution by carbon containing species in the air. For the graphene nanofluids lubrication as shown in Figure 9b, there were few scratches on the worn surface, and dark patches was formed during sliding process. The corresponding EDS analysis (spectrum B) was made to ascertain the ingredients. The strong signal of C element (28.2 wt.%) from EDS spectrum confirmed the presence of graphene on the worn surface. The graphene protective layer can smooth the surfaces and thus reduce friction coefficient and wear rate. However, graphene sheets stacked upon each other and showed no effective uniform coverage on the worn surface, and thus the lubricant performance of graphene sheets as a lubricant additive was limited. Compared with graphene nanofluids, it was clear that GO platelets were homogeneous distributed on the contact surface and that the surface had nearly none of scratch traces observed in Figure 9c. Moreover, the content of C element on the worn surface was up to 55.3 wt.% (spectrum C), which was almost 2-fold higher than that shown in spectrum B. It can be reasonably inferred that GO in dispersed water is able to form a block deposition film with good coverage on the rubbing surfaces, which finally leads to the significant improvement in tribological performance of the pure water. The results are in good accordance with the previous findings in Figure 4 and Figure 5, further verifying the best anti-wear ability of GO nanofluids among all samples.

**Figure 10:**
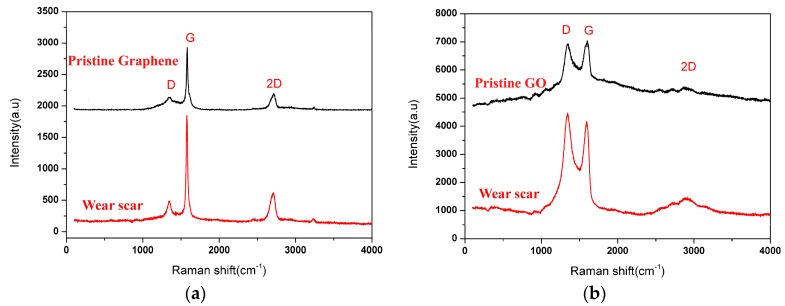
Raman results for the wear surface lubricated with (**a**) 0.5 wt.% graphene nanofluids; (**b**) 0.5 wt.% GO nanofluids (3 N, 0.08 m/s, 30 min).

Raman spectroscopy was carried out to further confirm that the excellent tribological performance is attributed to the presence of graphene or GO on the worn surface. Figure 10 exhibits the Raman spectrum measured in the wear track on the magnesium alloy surface lubricated with 0.5 wt.% graphene nanofluids and 0.5 wt.% GO nanofluids, compared with the corresponding spectrum of the pristine powder. According to Figure 10a, the Raman spectrum of the worn surface lubricated with graphene nanofluids exhibited three characteristic peaks of the D-band at 1350 cm^−1^, 2D-band at 2712 cm^−1^ and a G-band at 1580 cm^−1^, which are the typical Raman signals of graphene. This means that some graphene would be inlaid into the worn surface of the specimen to improve the tribological behaviors. However, the Raman spectrum for the deposited graphene on the worn surface is some different characteristics in comparison with the pristine powder. The intensity ratio of D to G band increased as compared to that of the pristine graphene powder, illustrating significant modification had occurred to graphene during sliding by converting it into the disordered graphitic structure. The GO on the worn surface was confirmed by the Raman spectra analysis of the two most intense features of the D band (1345 cm^−1^) and G band (1587 cm^−1^) as shown in Figure 10b. Similar to the graphene nanofluids lubricated surface, the GO on the worn surface also presented the strengthening of the intensity ratio of I_D_/I_G_, in contrast that of the pristine GO powder. This should not be a surprise considering that those flakes were lying in between the two sliding surfaces standing a pressure of several hundred MPa. Such phenomenon has been well documented in the previous reports [35,36].

### 3.6. Related Tribological Mechanism of Nanofluids

The tribological properties of potentially useful graphene and GO as water-based lubricant additives for magnesium alloy/steel contacts have been investigated in the present study. It is observed that both of the friction reduction and anti-wear ability of graphene or GO nanofluids were improved compared with those of pure water. Prior to any speculation of the friction-reducing and anti-wear mechanism, it is necessary to investigate the lubrication regime of the test situation. The lubrication regime in the tribological contacts can be divided into three categories, including boundary lubrication, mixed lubrication and hydrodynamic lubrication. The corresponding lubrication regime should be evaluated based on the value of *λ* in Equation (2), where *h*_min_ refers to the theoretical minimum film thickness separating the contact interfaces. The *h*_min_ can be calculated by the Hamrock-Dowson model displayed in Equation (3), and *R*_q_ is the combined surface roughness determined according to Equation (4) [37].

(2) $$\lambda =\frac{h_{\min}}{R_{q}}$$

(3) $$h_{\min}=2.8R^{\prime }{\left(\frac{\eta u_{e}}{E^{\prime }R^{\prime }}\right)}^{0.65}{\left(\frac{W_{y}}{E^{\prime }R^{\prime 2}}\right)}^{-0.21}$$

(4) $$R_{q=}\sqrt{R_{ball}{}^{2}+R_{flat}{}^{2}}$$

Of which *W*_y_ is the applied normal load, *u*_e_ is the linear velocity, *η* is the bulk viscosity of the nanofluids, *E*^’^ is the effective elastic modulus, and *R^’^* is the radius of the ball. On the basis of the material characteristics and test condition, the thickness of the lubricant film at the point of contact is 25, 20, 18, 16 nm for 1, 3, 5, 8 N, respectively. The corresponding *λ* values were confirmed to be 0.26, 0.21, 0.19, 0.17, respectively. The lubrication regime is usually defined by the following regulations: boundarylubrication (0.1 < *λ* < 1), mixed lubrication (1 ≤ *λ* ≤ 3), elastohydrodynamic lubrication (*λ* > 3) [38]. It is suggested that the test condition in this study is within the boundary lubrication regime.

The high friction generally occurs in the boundary lubrication regime where a beneficial fluid lubricant film is unable to form and separate the rubbing surfaces. Therefore, the addition of nanoparticles in the base media is essential to provide a surface protective layer. In this study, when 0.5 wt.% graphene is dispersed into water, the friction coefficient and wear rate are respectively reduced by 21.9% and 13.5% as compared to that of the benchmark water. We know that, during sliding process, graphene with water tends to penetrate into the interface of contact pairs and gradually accumulate on the magnesium alloy surface, resulting in the formation of a transfer films between the rubbing surfaces. This outlook was supported by the FESEM–EDS spectra and Raman imaging shown in Figure 9a and Figure 10a, respectively. Undoubtedly, the tribo-layer alleviates the metal-metal contact at the interface, thus reduces friction coefficient and wear rate [31,39]. Even so, further understanding the role of the protect film at disparate stage of the whole test is crucial and necessary. As shown in Figure 3, it is apparent that the friction coefficient for graphene nanofluids at the early stage of sliding has little difference from that of pure water. Similar evolution of friction coefficient with time for a steel-cast iron pairs lubricated by graphene nanofluids, where a higher friction coefficient at the beginning of the test can be observed, was reported by Dan Zheng et al. [36]. The authors explain that the high friction coefficient at the early stage of the tests could be attributed to the absent of tribo film on the contact region. Once the tribofilm generated, the friction coefficient gradually decreased. In addition, the graphene sheets stacked upon each other due to their poor dispersion in the water and showed no effective uniform coverage on the worn surface, and thus the friction-reducing and anti-wear behaviors of graphene sheets as a lubricant additive was limited.

In contrast, GO displayed higher friction-reducing and anti-wear capability than graphene did. The 0.5 wt.% addition of GO in the water offered reduction of friction coefficient by 77.5% and reduction of wear rate by 90% compared with the pure water, which is also larger than the reported values for other nanomaterial water-based additives, e.g., Al_2_O_3_ (27% friction and 22% wear reduction with the addition of 1 wt.%) [40], TiO_2_ nanoparticles (50% friction and 27% wear reduction with the addition of 0.8 wt.%) [41], and nanographite (44% friction and 49% wear reduction with the addition of 1.0 wt.%) [42]. It can be considered that the superior tribological performances of GO nanofluids results from the intrinsic surface oxygen-containing functionalized groups, such as carbonyl (C=O), hydroxide (C–OH) and carboxyl (COOH). Firstly, GO with the oxygen-containing functionalized groups, due to chemistry nature and polarity, favorably attach on the metal surface, resulting in the improved adhesion between the GO and the magnesium alloy. Therefore, GO shows a great advantage in achieving long-term stability and load bearing capacity compared with graphene as shown in Figure 6 and Figure 7. Secondly, the relatively low interfacial strength within the GO layers may have also contributed to the significant improvement in the tribological properties. The literature [43] demonstrated that the increase of the oxygen-containing functionalized groups in the pristine graphene lead to the degradation of the interlayer bond strength by molecular dynamics (MD) simulation. Also, the increase in the interplanar spacing between the GO layers may have increased the possibility of water molecules to be penetrated into the gap [44]. Thus, it was claimed that the low interfacial strength of GO layers and the water molecules in between the GO layers were responsible for the reduction of the shear strength during sliding. Thirdly, the oxygen-based groups arehydrophilic, resulting in excellent dispersion stability [45,46] and wetting property as shown in Figure 8. The superior dispersion stability of the GO in the water ensures its uninterrupted supply to the contact area followed the water. So the running-in time required to obtain very low friction values for GO nanofluids is shorter than graphene nanofluids as shown in Figure 3. Meanwhile, the excellent wetting property of GO nanofluids promotes the formation of the protective layer on the worn surfaces. This is supported by the EDS spectra (Figure 9), which shows that the content of C element on the GO nanofluids lubricated surface is nearly 2-fold higher than that of the graphene nanofluids lubricated surface. Even so, the acidity of the GO nanofluids could be concerned for metal lubrication. In this study, the pH value of the GO suspension measured by a digital calibrated pH meter was at 3.8, which is close to weak acid. In addition, no obvious corrosive wear on the worn surface as shown in Figure 9c. Therefore, the GO water-based nanofluids with the superior property can be readily available for potential industrial applications.

## 4. Conclusions

In this paper, the tribological behaviors of graphene or GO as water-based lubricant additives for magnesium alloy/steel contacts were evaluated by a reciprocating sliding ball-on-plate contact configuration. According to the analysis of the experimental results, the following conclusions can be drawn.

(1) Graphene and GO as water-based lubricant additives improved the friction-reducing and anti-wear abilities. The best tribological response of the magnesium alloy/steel pairs evaluated was obtained when graphene or GO at a concentration of 0.5 wt.% was added to water.
(2) Graphene and GO exhibited different friction-reducing and anti-wear efficiencies, and the tribological performances of GO are superior to that of graphene. Meanwhile, the positive effect of the GOnanofluids was also more pronounced in terms of the load-carrying capacity and the lubrication film endurance.
(3) The prominent lubricant performance of GO nanofluids can be attributed to the strong affinity between GO sheets and magnesium alloy surface, superior dispersion in the water and excellent wetting of the GO nanofluids on the magnesium alloy surface.

## Figures and Tables

**Table 1:** Mechanical properties of extruded AZ31 magnesium alloy used in this study.

Material	0.2% YS/MPa	UTS/MPa	Elongation/%	HV_0.01_
Extruded AZ31	142.1	305	18.5	66.7

**Table 2:** Testing conditions.

Test	Load (N)	Contact Pressure (MPa)	Sliding Speed (m/s)	Sliding Time (h)	Lubricants
The effect of concentration	3	312	0.08	0.5 h	Water Graphene nanofluids with different concentration GO nanofluids with different concentration
Carrying capacity	1	223	0.08	0.5 h	Water 0.5 wt.% Graphene nanofluids 0.5 wt.% GO nanofluids
	3	312			
	5	381			
	8	446			
Endurance of lubrication film	8	446	0.03	1.5 h	Water 0.5 wt.% Graphene nanofluids 0.5 wt.% GO nanofluids
